# Late Recognition of SARS in Nosocomial Outbreak, Toronto

**DOI:** 10.3201/eid1102.040607

**Published:** 2005-02

**Authors:** Thomas Wong, Tamara Wallington, L. Clifford McDonald, Zahid Abbas, Michael Christian, Donald E. Low, Denise Gravel, Marianna Ofner, Barbara Mederski, Lisa Berger, Lisa Hansen, Cheryl Harrison, Arlene King, Barbara Yaffe, Theresa Tam

**Affiliations:** * Public Health Agency of Canada, Ottawa, Ontario, Canada;; † University of Toronto, Toronto, Canada;; ‡ Toronto Public Health, Toronto, Canada;; § Centers for Disease Control and Prevention, Atlanta, Georgia, USA;; ¶ North York General Hospital, Ontario, Canada

**Keywords:** SARS, nosocomial infections, surveillance, dispatch

## Abstract

Late recognition of severe acute respiratory syndrome (SARS) was associated with no known SARS contact, hospitalization before the nosocomial outbreak was recognized, symptom onset while hospitalized, wards with SARS clusters, and postoperative status. SARS is difficult to recognize in hospitalized patients with a variety of underlying conditions in the absence of epidemiologic links.

Severe acute respiratory syndrome (SARS) spread globally in 2003, infecting >8,000 people and killing nearly 800. In total, 438 probable or suspected SARS cases and 44 deaths were reported in Canada (*1**,**2*). SARS was first recognized retrospectively in Canada in a woman who had returned from Hong Kong on February 23, 2003. This international connection ignited the outbreak in Canada, which affected mainly the Toronto area (*1**,**2*).

After enhanced infection control precautions and public health measures were implemented in March 2003, the Canadian outbreak began to subside in April. On May 14, the World Health Organization (WHO) took Toronto off the list as a SARS-affected area in the absence of newly reported cases for at least 2 incubation periods after the last SARS case-patient was isolated. In accordance with public health principles, the enhanced measures were selectively relaxed in low-risk settings in Toronto area hospitals in early May 2003, although full precautions were still recommended for patients with febrile respiratory illnesses. In the third week of May, a cluster of febrile respiratory illness at a Toronto area rehabilitation hospital was reported to the health department. Traceback of these SARS cases identified the index patient as a postoperative patient who was transferred from hospital X to the rehabilitation hospital. This link uncovered clusters of unrecognized SARS infections on a surgical ward and a psychiatry ward at hospital X. Investigation determined that the ventilation system did not contribute to the spread of SARS at that hospital. On May 23, 2003, hospital X was closed to nonobstetric admissions other than newly identified SARS cases, and SARS precautions were reintroduced. As part of an outbreak investigation, we explored potential factors contributing to the late recognition of SARS infections in a cohort of persons with SARS admitted to hospital X.

## The Study

Hospital X is a Toronto-area community hospital with 425 beds. During the 2003 outbreak in Toronto, dedicated SARS inpatient units were created at the hospital. All nonhealthcare workers with probable or suspected SARS, according to the WHO case definition (*3*), exposed at and admitted to hospital X with symptom onset from April 17, 2003, to June 8, 2003, were included in this retrospective cohort investigation. Healthcare workers were excluded. If SARS was not recorded as a possible diagnosis in the medical chart, despite SARS-defining manifestations for at least 24 hours of hospitalization, recognition of SARS was classified as late. Otherwise, recognition was classified as prompt. Laboratory diagnosis of SARS was obtained by reverse transcriptase–polymerase chain reaction (RT-PCR) or serologic testing (*4**,**5*).

SPSS version 11.0 (SPSS, Inc., Chicago, IL, USA) was used for statistical analysis. Continuous variables were dichotomized about the median. Factors associated with late recognition were deemed statistically significant if the p value was <0.05 (2-tailed), using chi-square test with Yates correction or Fisher exact test, when appropriate. Relative risks with 95% confidence intervals were listed as undefined when certain cell sizes were 0. The small sample size, small number of patients with the outcome of interest (late SARS recognition), and small cell size for some dichotomous variables precludes multivariate logistic regression analysis.

The SARS outbreak involved 88 case-patients whose exposure setting was hospital X (Figure). SARS occurred in 50 patients, family members, or visitors. Thirty-three of these 50 persons were admitted to hospital X, and all 33 were included in this analysis.

**Figure:**
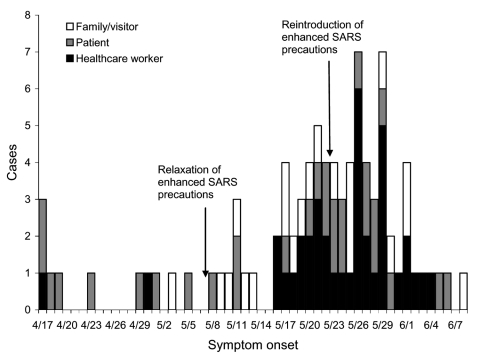
Reported probable and suspected severe acute respiratory syndrome cases in persons (or their family members) with symptom onset after April 17, 2003, whose exposure setting was hospital X.

SARS-associated coronavirus (SARS-CoV) laboratory results were available for 29 (88%) of the 33 SARS patients. No samples were available for SARS-CoV testing from 4 deceased patients. Twenty-four (83%) of the 29 SARS patients tested were positive by RT-PCR, serology, or both. The 5 remaining patients had negative acute-phase SARS-CoV serologic results, and their convalescent-phase results were not available (data not shown).

Eleven (33%) patients had late recognition of SARS. Their mean age was 68.8 years, and 8 (73%) were postoperative patients. All were admitted before May 23, 2003, the day when Hospital X reintroduced enhanced SARS precautions. No casepatients had a recognized close contact with another SARS patient initially. None were travel related. (Table 1). Six (55%) patients were admitted to an intensive care unit (ICU), and 3 (27%) required mechanical ventilation (Table 2). All patients had infiltrates on chest radiographs; infiltrates of 9 (81%) were bilateral.

**Table 1:** Proportion of patients with late SARS recognition by demographic and exposure characteristics, Toronto, hospital X, April 17–June 8, 2003*

Characteristics	Late SARS recognition (%)	RR (95% CI)	p value
Age (y)		0.6 (0.2–1.7)	0.5
<58.5	4/16 (25.0)		
>58.5	7/17 (41.2)		
Sex		0.5 (0.1–1.4)	0.3
F	3/15 (20.0)		
M	8/18 (44.4)		
Aware of close contact with a SARS patient		UD	< 0.001
Yes	0/14 (0)		
No	11/19 (57.9)		
Exposure from being an inpatient on wards at hospital X with SARS clusters		4.1 (1.3–12.7)	< 0.01
Yes	8/13 (61.5)		
No	3/20 (15.0)		
Admitted to hospital X before May 23, 2003		UD	< 0.01
Yes	11/20 (55.0)		
No	0/13 (0)		
SARS symptom onset while hospitalized		UD	< 0.001
Yes	11/15 (73.3)		
No	0/18 (0)		

*SARS, severe acute respiratory syndrome; RR, relative risk; CI, confidence interval; F, female; M, male; UD, undefined.

**Table 2:** Proportion of patients with late SARS recognition by clinical characteristics, Toronto, hospital X, April 17–June 8, 2003*

Characteristics	Late SARS recognition/total SARS (%)	RR (95% CI)	p value
Postoperative		5.3 (1.8–16.2)	0.001
Yes	8/11 (72.7)		
No	3/22 (13.6)		
Maximum temperature during hospitalization (°C)		0.8 (0.3–3.1)	1.0
<38.8	5/15 (33.3)		
>38.8	6/15 (40.0)		
First symptom includes			
Fever		1.0 (0.2–5.4)	1.0
Yes	10/30 (33.3)		
No	1/3 (33.3)		
Cough		0.7 (0.2–2.6)	0.7
Yes	2/8 (25.0)		
No	9/25 (36.0)		
Dyspnea		0.4 (0.1–2.4)	0.4
Yes	1/7 (14.3)		
No	10/26(38.5)		
Diarrhea		0.6 (0.1–3.5)	0.6
Yes	1/5 (20.0)		
No	10/28 (35.7)		
Nausea/vomiting		3.2 (1.9–5.4)	0.3
Yes	1/1 (100)		
No	10/32 (31.3)		
Admitted to ICU		1.4 (0.5–3.8)	0.7
Yes	6/15 (40.0)		
No	5/18 (27.8)		
Supplemental oxygen		5.0 (0.7–34.2)	0.054
Yes	10/22 (45.5)		
No	1/11 (9.1)		
Mechanical ventilation		0.8 (0.3–2.3)	0.7
Yes	3/11 (27.3)		
No	8/22 (36.4)		
Death		1.3 (0.5–3.5)	0.7
Yes	4/10 (40.0)		
No	7/23 (30.4)		
Treatment with			
Ribavirin		UD	1.0
Yes	0/2 (0)		
No	10/30 (33.3)		
Corticosteroids		0.3 (0.1–1.1)	0.06
Yes	2/15 (13.3)		
No	8/17 (47.1)		
Antimicrobial drugs		1.0 (0.3–3.0)	1.0
Yes	8/24 (33.3)		
No	3/9 (33.3)		

*SARS, severe acute respiratory syndrome; RR, relative risk; CI, confidence interval; UD, undefined; ICU, intensive care unit.

Using univariate analysis, we found that patients with late recognition of SARS were more likely to have no known contact with another SARS patient (p < 0.001), to have been a patient on a ward where SARS cluster occurred (p < 0.01), to be admitted before the nosocomial outbreak was recognized at the hospital (p < 0.01), to have symptom onset while hospitalized (p < 0.001), and to be a postoperative patient. (p = 0.001) (Tables 1 and 2). Clinical findings and laboratory abnormalities during hospitalization were not associated with late SARS recognition. The small sample size, small number of patients with late SARS recognition, and small cell size for some dichotomous variables precluded multivariate logistic regression analysis.

The hospital reintroduced enhanced SARS precautions on May 23, 2003, under the direction of public health authorities promptly after the nosocomial outbreak was recognized. For patients admitted before that date (N = 20), the relative risk for late recognition of SARS for postoperative patients was 2.7 (95% confidence interval 0.99–7.2, p = 0.07) and was just short of statistical significance (data not shown). Once SARS transmission was recognized at hospital X and enhanced infection control precautions were reinstituted, clinicians were more likely to suspect SARS, and nosocomial transmission was ended abruptly.

## Conclusions

Our results highlight the difficulty clinicians can have in recognizing locally acquired SARS among patients with other underlying medical conditions but with no apparent epidemiologic linkage. The patients had no known contact with other SARS patients, did not have a travel history to SARS-affected areas outside of Canada when the outbreak was thought to be over in Toronto, and were unaware of a simmering outbreak associated with hospital X. Perhaps because of the nonspecific nature of clinical manifestations, SARS can be especially difficult to recognize among patients already hospitalized for other reasons. These symptoms overlap many of the symptoms of hospitalized febrile postoperative patients and patients with other respiratory illnesses (*6**–**12*). In addition, at the time of this nosocomial outbreak, the full spectrum of the clinical signs and symptoms of SARS had not yet been well characterized (*11*).

To detect SARS early, health professionals need to look not only for epidemiologic links but also clusters of unexplained respiratory infection. A cluster of respiratory infections among families and visitors may not be evident initially because hospitals do not normally track infections in inpatients' families and visitors. In addition, infected patients may be asymptomatic before they are transferred to another healthcare facility.

Even though our investigation generated interesting hypotheses, it had several limitations, which included reliance on retrospective chart reviews to abstract data. Such information may have been affected by missing data or recall bias. Our analysis did not include hospital workers, nor did it include persons who were exposed at hospital X but were subsequently admitted elsewhere. Studies that include hospital workers and persons admitted to other hospitals are needed. Seroprevalence studies will be helpful because some infected persons may not be symptomatic. The small sample size limited the power to detect small differences and precluded multivariate analysis. A small number of patients with a diagnosis of SARS may not actually have been infected with SARS-CoV. This possibility could have biased the results either way. However, this number is likely small because most case-patients tested positive for SARS-CoV.

Although our results highlight the challenge of recognizing SARS among hospitalized patients, the occurrence of seasonal respiratory infections such as influenza may further compound the difficulty in identifying a SARS case. In places where recent SARS transmission has occurred, SARS should be considered during the evaluation of nosocomial as well as community-acquired pneumonia. This recommendation is particularly important if the hospital housed SARS patients within the previous 20 days or if unexplained febrile respiratory clusters had been noticed within the institution.

This nosocomial outbreak underscores the importance of sharing information among clinicians, laboratories, infection control departments, occupational health services, and public health departments and of collaborating seamlessly in the search for clusters of respiratory infections. A sensitive infectious disease surveillance system operated by the infection control officer must be in place in healthcare facilities for early detection and implementation of appropriate measures to interrupt transmission (*13**,**14*). This surveillance should include the monitoring of increased absenteeism among healthcare workers; unusual fever or pneumonia clusters among patients, family, visitors, and healthcare workers; pneumonia deaths; and laboratory testing for respiratory pathogens or SARS-CoV. These steps require commitment, preparedness planning, resources, and training. The lack of a rapid, sensitive, and specific diagnostic test for early SARS infection hampers the ability of clinicians to make a prompt diagnosis in cases when an epidemiologic link is missing (*15*).
